# Lithium battery reusing and recycling: A circular economy insight^☆^

**DOI:** 10.1016/j.heliyon.2019.e01866

**Published:** 2019-06-15

**Authors:** Mario Pagliaro, Francesco Meneguzzo

**Affiliations:** a Istituto per lo Studio dei Materiali Nanostrutturati, CNR, via U. La Malfa 153, 90146, Palermo, Italy; b Istituto di Biometeorologia, CNR, via G. Caproni 8, 50145, Firenze, Italy

**Keywords:** Energy, Lithium-ion battery, Battery recycling, Battery electric vehicle, Circular economy

## Abstract

Driven by the rapid uptake of battery electric vehicles, Li-ion power batteries are increasingly reused in stationary energy storage systems, and eventually recycled to recover all the valued components. Offering an updated global perspective, this study provides a circular economy insight on lithium-ion battery reuse and recycling.

## 1 Introduction

Driven by the electric vehicle (EV) boom [1], which led to a 3-fold increase in the price of lithium [2] and a 4-fold increase in that of cobalt [3] between 2016 and 2018, reclaiming lithium, cobalt, manganese and nickel (along with other valued materials like copper, aluminum and graphite) from spent lithium ion batteries has lately become profitable. Perhaps not surprisingly, numerous new lithium battery recycling plants started operation across the world, and those existing are expanding capacity.

In about 2 years, the recycling of lithium batteries which still in 2016 was claimed in Europe to lack economic viability as “only 3% of the material mix in batteries is made of lithium” [4], became profitable and convenient.

Investments started to flow targeting opportunities not only for recycling but also for refurbishing and reusing retired EV lithium-ion batteries (LIBs) in energy storage systems. “Certain companies” reads a working document of the European Commission dated mid 2018, “have already begun investing in recycling of used EV batteries in Europe (e.g. in Belgium and in France). Some have teamed up with car manufacturers to collect and recycle batteries” [5].

Hence, as lately emphasized by Melin, a reputed consultant in lithium-ion battery life cycle, “when it is not rare to read about recycling rates of 3 or 5 per cent… 58 per cent will be recycled this year” [6].

The environmental and economic benefits of LIB recycling are significant. As the lithium-ion recycling industry consolidates and the demand for spent LIBs increases, the old practice for which small batteries used by portable electronic devices were hazardously stockpiled in generic materials recovery facilities causing fires due to thermal runaway from damaged or short circuited batteries [7], will become a thing of the past.

This trend, we argument in this study, will further evolve and eventually first generation LIB recycling processes will be replaced by green chemistry processes producing highly pure (“battery-grade”) lithium, cobalt and manganese compounds along with graphite, copper and aluminum.

Recycling, in general, relies on first generation recovery technologies in which a physical treatment to obtain different streams of raw materials is followed by a hydrometallurgical process (leaching and extraction) to extract metals [8].

Lithium-ion batteries, indeed, generally use a graphite anode and a cathode made of lithium metal oxides generally comprised of lithium-iron phosphate (LFP), lithium-nickel manganese cobalt (NMC), lithium nickel cobalt aluminum oxide (NCA), lithium-manganese oxide (LMO), or lithium-titanate oxide (LTO). First generation LIBs mainly used in portable electronics used lithium-cobalt oxide (LCO) [9].

The battery cells are assembled in modules and modules further assembled in battery packs. The voltage from “power” batteries supplying current to the motor of electric passenger cars or buses, can respectively top 300 V or even exceed 600 V.

Offering an updated global perspective, this study provides a circular economy insight on lithium-ion battery reuse and recycling.

## 2 Main text

### 2.1 Technology and chemistry aspects

By weight percentage (g material/g battery), a typical lithium-ion battery comprises about: 7% Co, 7% Li (expressed as lithium carbonate equivalent, 1 g of lithium = 5.17 g LCE), 4% Ni, 5% Mn, 10% Cu, 15% Al, 16% graphite, and 36% other materials [10].

Besides so called “calendar ageing”, a lithium-ion battery becomes “spent” (reduced ability to store and deliver electricity) mainly because during the charge and discharge cycles taking place in the battery cells a solid product forms due to reaction of the lithiated anode with the alkyl carbonate comprising the electrolyte solution [11].

The resulting solid electrolyte interphase mainly consisting of stable (such as Li_2_CO_3_) and metastable components (polymers, ROCO_2_Li, (CH_2_OCO_2_Li)_2_, and ROLi prone to decompose exothermically at >90 °C, releasing flammable gases and oxygen) [7] progressively deposits on the anode surface forming a passivating film. This film limits the electrochemical reaction by making graphite sites inaccessible for Li^+^ to intercalate and thus leading to an increase in internal ohmic resistance.

A typical EV lithium ion battery pack has a useful first life of 200,000–250,000 km ​[12], even though increasingly adopted fast-charging at >50 kW reduces the battery pack duration since battery degradation rapidly accelerates with charging current [13].

When, the automotive battery pack loses 20% (15% for certain EV models) of its initial capacity it becomes unfit for traction as the lower capacity of battery affects acceleration, range and regeneration capabilities of the vehicle [14].

#### 2.1.1 Second-life batteries

Besides the beneficial effect on the price of grid electricity due to the concomitant expansion of EVs utilization and renewable energy generation (particularly solar photovoltaics) ​[15], a second synergistic effect of battery electric vehicle on renewable electricity uptake lies in the possibility to reuse the batteries at the end of their automotive lifecycle for stationary energy storage, nicely fulfilling the key “refurbish, reuse, recycle” circular economy principle.

Compared to use in EVs, stationary applications demand lower current density from the battery pack. Hence, batteries retaining between 80-85% of their original capacity are collected. Battery modules found to have similar power and life are sorted out and re-assembled in new “repurposed” battery packs, ready for stationary usage ​[16], such as utility-scale grid, building and telecommunication tower storage.

**Fig. 1:**
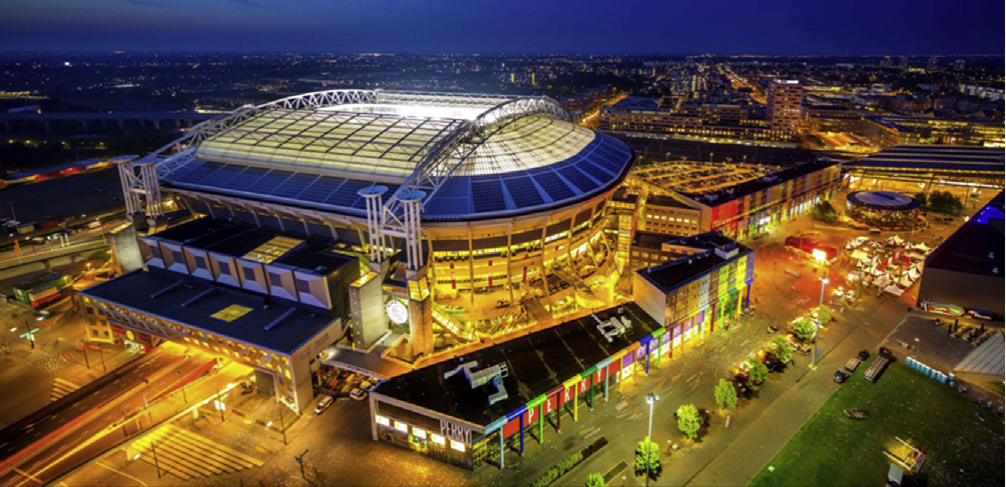
Amsterdam's “Johan Cruyff Arena” multipurpose stadium. [Photo courtesy of Eaton].

A significant public demonstration of the ability of repurposed batteries to provide energy storage and grid services (regulation of the alternating current frequency in the grid) is the 3 MW (nominal power)/2.8 MWh (nominal capacity) energy storage system installed in 2018 at Amsterdam's “Joahn Cruyff Arena”, (Fig. 1) [17].

During events at the stadium, the demand for electricity lighting, powering broadcasting, information technology equipment, catering, and security services increases from a baseload of around 200 kW to more than 3000 kW, for the entire duration of the event [17].

The new energy storage system installed at Amsterdam's Arena is comprised of 590 battery packs (340 new and 250 second-life batteries originating from EV 24 kWh battery packs whose original capacity is now slightly less than 20 kWh).

Directly supplied by the EV maker the second-life batteries are certified to last 10 years, namely the equivalent of batteries included in 148 used exemplars of the first generation world's best selling EV [18].

**Fig. 2:**
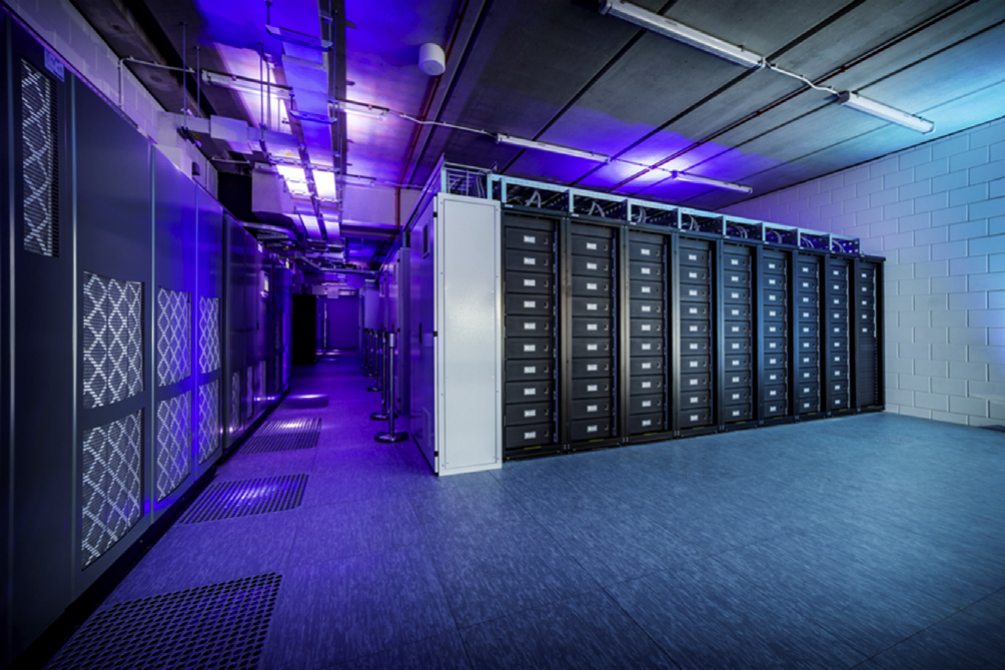
Racks with part of the 2.8MWh energy storage system at the “Johan Cruyff Arena”. [Photo courtesy of Eaton].

The batteries are contained in 61 battery racks (Fig. 2). Four bi-directional inverters manage the energy flows from the 4,200 rooftop PV modules, from and to the grid, and from the batteries to the stadium loads and to the grid (we remind that the grid accepts and supplies only alternating current, whereas the PV modules and the batteries supply direct current only).

The new energy storage system enables optimal use of both solar PV and grid electricity retrieved at low cost from the grid during the night hours.

Now the PV energy generated during the day, rather than being fed into the grid and sold to the grid operator at low price, goes to charge the 2.8 MWh battery pack whose nominal capacity was chosen to meet the energy demand from the stadium loads for 1 h during the most important events with maximum power absorption; and for 3 h when accessory services such as catering are not in use [17].

Flattening (“shaving” in the jargon used by electricity practitioners) the peak demand with free PV or low cost grid electricity stored in the lithium batteries *i*) cuts the diesel fuel cost (fuel is used in generators whose use is made compulsory from football authorities), *ii*) avoids peak demand charges, and *iii*) generates a revenue stream when the energy storage system is used to provide well paid grid-balancing services, such as frequency control.

Similar energy storage systems combining second-life EV battery modules with battery and power management digital technology for both residential, commercial and industrial applications are increasingly commercialized across the world by a number of companies.

Similarly, in China the world's biggest operator of telecommunication towers, since 2018 ended purchase of lead-acid batteries. All existing and rapidly ageing lead-acid batteries currently installed for back-up power at 98% of its 2 million telecom tower base stations (54 GWh battery storage demand) will be replaced by second-life LIBs [19]. Partnership agreements were signed with more than 16 EV and battery manufacturers, as second-life LIBs in 2018 were reported to be priced at less than $100/kWh, namely the same price of new lead-acid batteries [19].

For comparison, this translates into forthcoming demand for up to 2 million retired EV batteries only from China's telecom base station back-up, since one single tower needs about 30 kWh back-up battery [19].

According to a thorough analysis conducted in 2017 by Melin, by 2025 about 75 per cent spent EV batteries will be reused in second-life solutions for several years after retirement from vehicles, after which they will be sent to recycling to recover all the valued components [20].

#### 2.1.2 New green chemistry technologies

Reviewing first-generation metal recovery processes using pyrometallurgical or hydrometallurgical methods, scholars in China lately emphasized the need for new “selective leaching of most of the valuable metals from the spent LIBs” [8].

Discovered in 2015, one such green process for the recovery of metals from spent Li-ion batteries makes use of citric acid (H_3_Cit) and aqueous H_2_O_2_ affording Co and Li in excellent recovery yields (98% Co and 99% Li) [21].

**Fig. 3:**
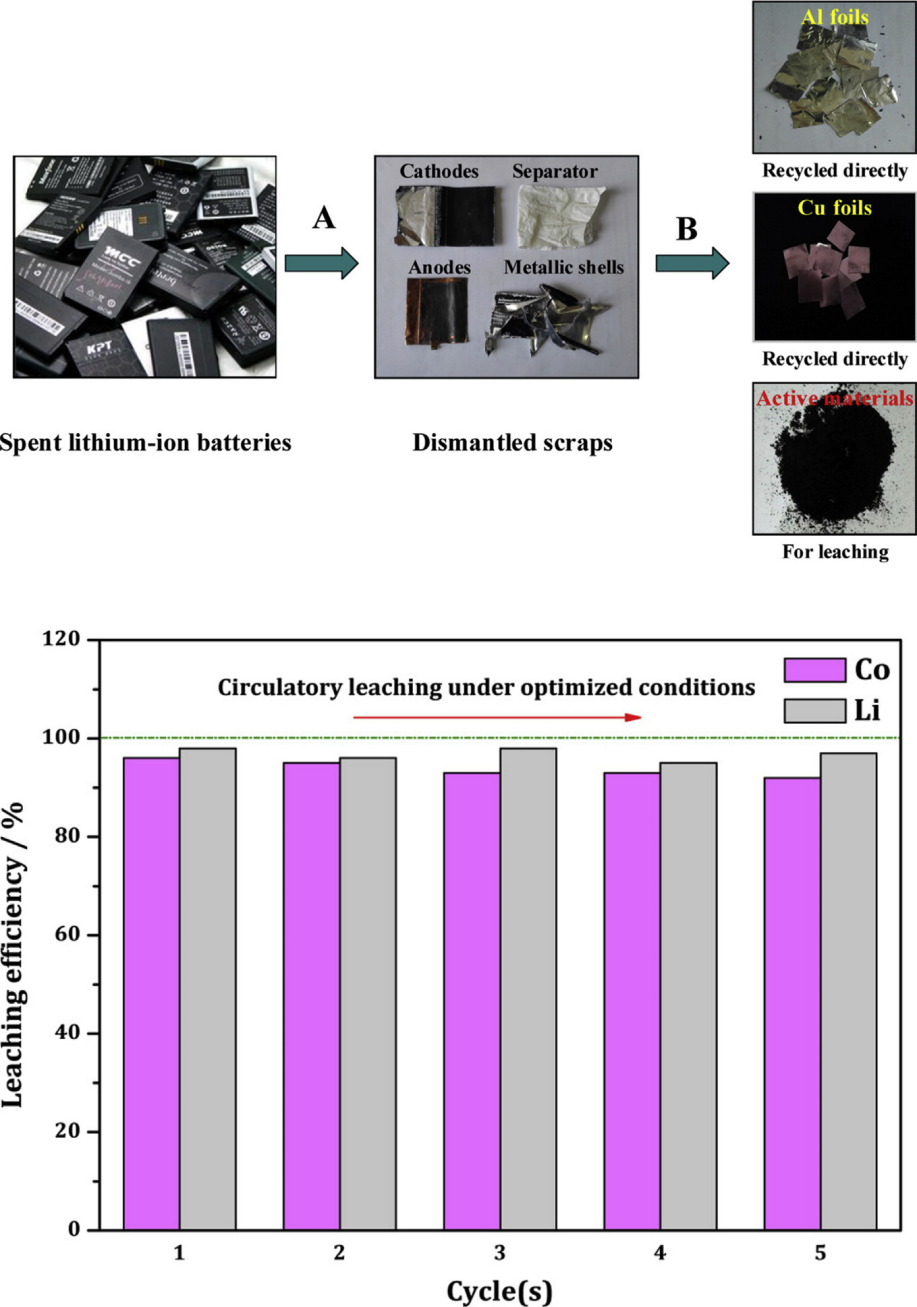
Simplified pretreatment process of spent LIBs based on citric aicd/hydrogen peroxide oxidative leaching of Co and Li: (A) manual dismantling; (B) peeling off Al/Cu foils and recycling of Al and Cu (*top*); and circulatory leaching experiments under the optimized conditions using citric acid and H_2_O_2_ (*bottom*). [Reproduced with permission from Ref.21, Copyright American Chemical Society].

In detail, the spent batteries are first discharged and then manually dismantled to recover the Al and Cu foils in metallic form and the separator, directly recycled after dismantling (Fig. 3).

The waste cathode materials ground into finer fractions for the subsequent extraction process is obtained by calcining at 700 °C for 2 h the cathode materials to remove carbon.

The powder of cathode material thereby obtained is used as raw material for the leaching process under optimized and mild extraction conditions (80 min, 70 °C, 2.0 M H_2_O_2_, with reductant dosage of 0.6 wt%, and slurry density of 50 g/L).

Aqueous H_2_O_2_ acts as clean reductant during metal leaching (Eq. 1), with both metal ions and waste citric acid being simultaneously recovered by selective precipitation (unbalanced).

(1) H_3_Cit + LiCoO_2_ + H_2_O_2_→ Co_3_(Cit)_2_ + Co(HCit) + Co(H_2_Cit)_2_ + Li_3_Cit + Li_2_(HCit) + Li(H_2_Cit) + H_2_O + O_2_

Co and Li ions dissolved in the lixivium are treated with oxalic acid and phosphoric acid solutions to recover Co and Li. The total reaction equation (Eq. 2) shows that water and oxygen are the only byproducts in the whole recovery process (unbalanced).

(2) LiCoO_2_ + H_2_O_2_ + H_2_C_2_O_4_ + H_3_PO_4_→ Co(C_2_O_4_)_2_ + Li_3_(PO)_4_ + H_2_O + O_2_

In a truly closed-loop route typical of the circular economy, about 99% Co and 93% Li could be recovered as CoC_2_O_4_·2H_2_O and Li_3_PO_4_, respectively, whereas the recycled citric acid shows similar leaching capability as fresh acid (Fig. 3, bottom).

LFP batteries, we have discussed elsewhere ​[1], will remain for many years the dominating lithium battery technology used by electric vehicles. It is therefore particularly relevant the recent discovery of a green and economically viable process for recycling entire spent LiFePO_4_ batteries to battery grade (99 wt%) Li_2_CO_3_ ready for manufacturing new LFP batteries [22].

The process is based on the selective leaching of lithium based on oxidation of LiFePO_4_ to FePO_4_ with aqueous sodium persulfate (Na_2_S_2_O_8_), forcing lithium deintercalating from the cathode (Eq. 3), while neither Fe (0.048% leaching) nor P (0.387%) leach out from the cathode structure whose olivine crystal structure is fully retained during the lithium leaching process.

(3) 2LiFePO_4_ + Na_2_S_2_O_8_ → 2FePO_4_ ↓ + Li_2_SO_4_ + Na_2_SO_4_

**Fig. 4:**
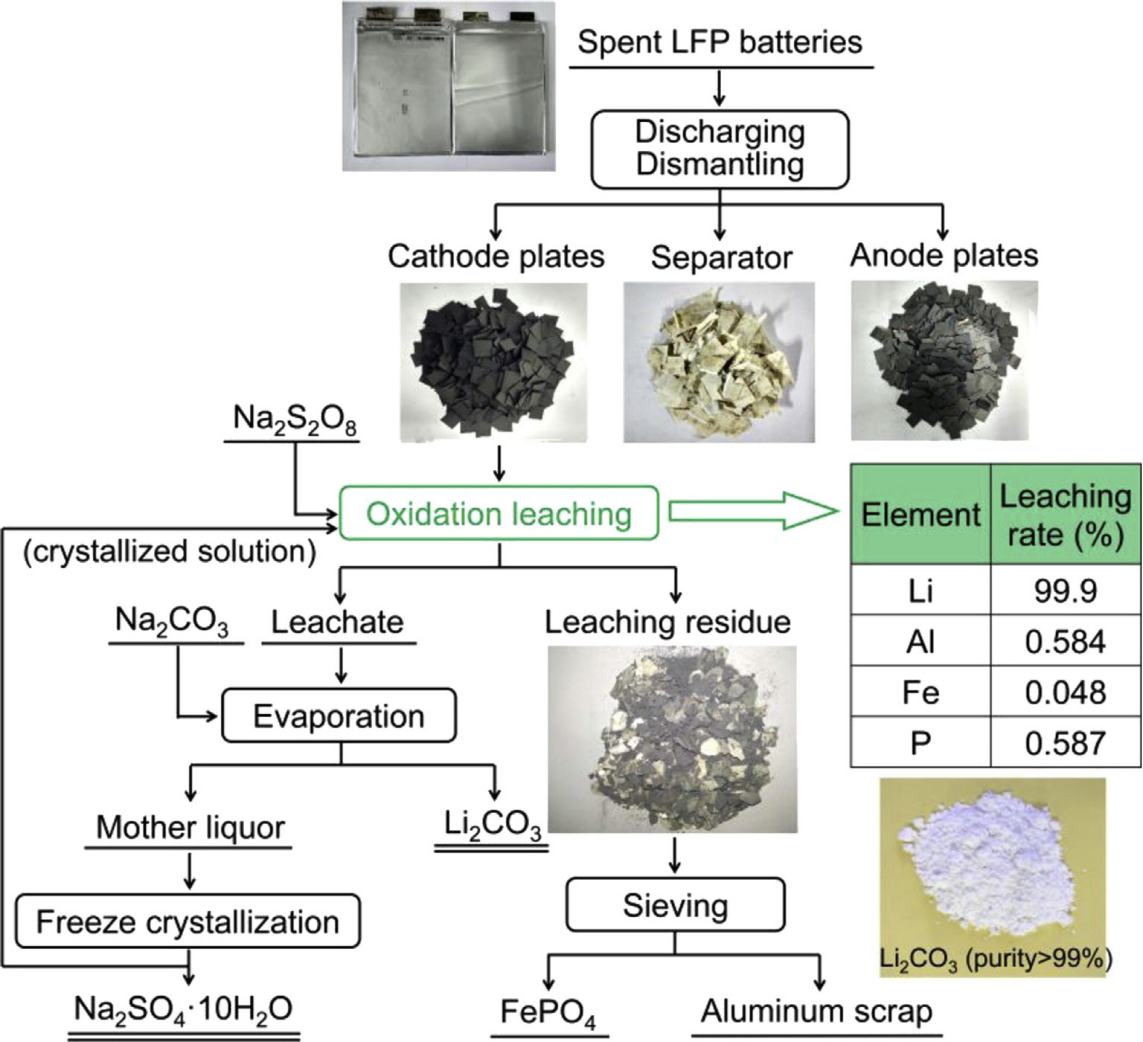
Flowsheet of the method for treating entire spent LiFePO_4_ batteries using aqueous sodium persulfate under neutral conditions for Li leaching. [Reproduced with permission from Ref.22, Copyright American Chemical Society].

In detail (Fig. 4), cathode scrap of LFP powder attached on Al foil obtained by a local battery recycling company is first separated from soft-package batteries via discharging and dismantling, and then cut into small pieces.

More than 99% of Li is leached without the addition of acid and alkali under the optimal conditions of 1.05 times the stoichiometric amount of persulfate, under remarkably mild conditions (25 °C, 20 min stirring a 300 g L^−1^ suspension of powdered cathode plates) with nearly no wastewater and solid waste generation.

No prior separation of cathode active material and Al foil (the most demanding procedure in the present recycling process of spent LIBs) is required because in the strong oxidative environment, Al is passivated (formation of a thin layer of Al_2_O_3_) resulting in an extremely low leaching of Al.

The leaching of lithium is very rapid with >90% of Li leached into the solution in only 5 min, an further increases to 99.8% by prolonging the leaching time to 20 min. As a result, the most valuable element in spent LFP batteries is directly recovered as Li_2_CO_3_ of high purity (>99%) by simple addition of Na_2_CO_3_ to the leachate followed by evaporation.

In a closed-loop method with great potential for industrial upscale, the mother liquor obtained after evaporation is used to prepare valued Na_2_SO_4_·10H_2_O product via freeze crystallization, whereas the crystallized solution is returned to leach another batch of raw cathodes with the addition of fresh Na_2_S_2_O_8_.

Besides Li recovery, the process enables direct cathode recycling to make new LFP cathodes for new batteries using the well-crystallized orthorhombic FePO_4_ leaching residue whose XRD pattern is close to that of raw LiFePO_4_ cathodes (the reverse of the phase transformation occurring in the charging of LFP battery, in which LiFePO_4_ releases Li^+^ ion and turns into FePO_4_).

Affording highly pure (>99.5%) lithium carbonate, the aforementioned processes solve the main problem which so far has limited the industrial uptake of green chemistry processes in LIB recycling, namely the “lengthy processing and purification processes of the raw materials to reach battery grade” [23] which determines “the true cost to manufacture” [23] Li-ion batteries.

Practically useful research in the field of green chemistry recycling processes continues at fast pace.

Selected examples include the simultaneous recovery of Li and Co from LiCoO_2_ cathode materials in a single step with good leaching efficiency (97% for Li and 98% for Co) using 0.6 M tartaric acid as leaching agent and 3% (v/v) H_2_O_2_ as reducatant (30 min at 80 °C with a solid to liquid ratio of 30 mL/g) ​[24]; and the recovery of all valuable metals from LiNi_0.5_Co_0.2_Mn_0.3_O_2_ cathode with excellent leaching efficiency (100% for Li, 93.38% for Ni, 91.63% for Co, and 92.00% for Mn) using an environmentally friendly leachant mixture of 0.2 M phosphoric acid and 0.4 M citric acid [25].

In the latter case, acid consumption is low, and the extraction time short (30 min at 90 °C with a solid to liquid ratio of 20 g/L) with no need for reductant as citric acid acts both as leachant and reductant.

### 2.2 Economic aspects

In a recent patent [10], seven main components (cobalt, lithium, copper, graphite, nickel, aluminum, and manganese) were reported to comprise >90% of the economic value of a spent lithium-ion battery: Co (39%) and Li (16%, as LCE equivalent) followed by Cu (12%), graphite (10%), Ni (9%), Al (5%) and Mn (2%).

The economic (and environmental) advantages of EVs are so large and significant (electric buses, for example) [26] that, regardless of rapidly growing output from new large factories in China, the demand of Li-ion batteries currently overcomes supply. This is especially the case for countries and regions like Europe where limited Li-ion battery manufacturing takes place. One Germany's bus manufacturer, for example, by early 2019 was reported to be unable to get the batteries needed to start manufacturing electric buses in 2020 [27].

New regulation in China now holds EV makers responsible for the recovery of batteries, requiring them to set up recycling channels and service outlets where old batteries can be collected, stored and transferred to recycling companies. By the end of February 2019, 393 carmakers, 44 scrapped car dismantling enterprises, 37 cascade utilization enterprises and 42 recycling enterprises had already joined the new traceability platform to track origin and owners of discarded batteries [28].

Furthermore, since 2017 new legislation forbids to import in China electronic waste, including batteries, which is leading China-based companies formerly supplying lithium carbonate, cobalt and nickel sulfates obtained from batteries retired from large consumer electronics manufacturers to establish new recycling plants “overseas” (in South Korea for example) ​[29]; as well as foreign EV battery makers to open recycling plants in China [30].

**Fig. 5:**
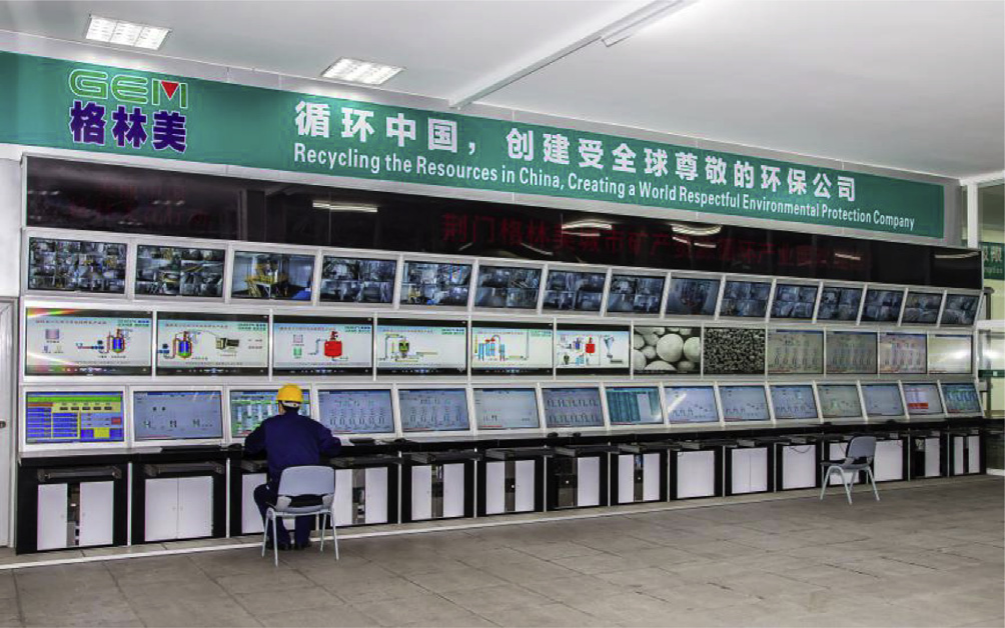
The automatic control system of GEM power battery precursor material production line. [Photo courtesy of GEM Co., kindly reproduced from gem.com.cn].

Industrial LIB recycling companies in China include Taisen Recycling, Zhejiang Huayou Cobalt, Brunp, Jinqiao Group, Jiangxi Ganfeng Lithium and GEM. The latter company, for example, operates in China 13 automated battery dismantling and recycling facilities where it manufactures the cathode precursors (Fig. 5), with an annual production capacity of cobalt, nickel materials of lithium ion batteries and cathode material exceeding 50,000 tons [31].

The products resulting from battery recycling are sold to battery manufacturers. Hence, it may not be surprising to learn that large battery manufacturers own recycling companies, as in the case of Brunp, a lithium-ion battery recycler in Hunan.

Though smaller, from Singapore (TES-AMM operating a plant using an hydrometallurgical process developed in France), through South Korea (SungEel) and Belgium (Umicore), from the U.S. and Canada (Retriev Technologies) through Australia (Envirostream Australia) and Great Britain (Belmont Trading), several other companies across the world operate LIB recycling facilities.

The list above is far from being exhaustive. What is relevant here is that, driven by dramatically growing uptake of LIBs for electric vehicles, recycling companies are rapidly expanding their facilities and new companies are entering the market. For instance, by early 2019 when the company recycled over 8,000 tons of retired batteries annually through an hydrometallurgical process, a Korean firm was undergoing a 5-fold expansion with three new plants due to start operations in Hungary, India, and in USA [32].

Market forecasts for the LIB recycling industry agree on significant growth, though forecasted figures vary. According to the aforementioned 2017 report ​[6, 33], recycled lithium will reach 9 percent of total lithium battery supply in 2025 (namely 5,800 tonnes of recycled lithium, or 30,000 tonnes LCE), and that of cobalt almost 20 percent of the demand, with >66% lithium-ion batteries being recycled in China.

New green chemistry technology will further contribute to lower recycling costs. Amid the numerous green recovery processes, the battery recycling industry will uptake the cheapest and most versatile.

Indeed, upon developing the leaching process based on citric acid/phosphoric acid ​[25], Zhou and co-workers compared it with two other efficient and rapid metal recovery processes in leaching LiNi_x_Co_y_Mn_z_O_2_ cathode material, namely those using lactic [34] acid and hydrogen peroxide, and malic [35] acid and hydrogen peroxide.

The three processes have similar leaching temperature, solid to liquid ratio and leaching time. Assuming therefore to treat one ton of waste cathode materials, the difference in cost mainly stems from raw material prices, with the cost of the phosphoric acid/citric acid leaching process about 30% lower than the malic acid process, and approx. 38% lower than that using expensive lactic acid [25].

Low cost citric acid, indeed, is the single largest chemical obtained via biomass fermentation and the most widely employed organic acid [36].

## 3 Conclusions

In early 2017, Zhao published one of the first comprehensive books on the reuse and recycling of lithium-ion batteries [37]. Referring to the “uncertain performance and service life of retired power batteries”, ending the “Market Development of Reuse and Recycling of Power Batteries” chapter, he wrote:

“The profit margin in the reuse of lithium-ion power batteries is unclear. Although data on batteries provided by lithium-ion power battery producers state that the batteries removed from new energy vehicles retain 70–80% valid energy and appear competitive in costs, there are still many challenges when energy storage is focused in the field of battery reuse” [38].

Two years later, a large electric power company started construction of a 268.6 MWh energy storage plant in the east China's Jiangsu Province. The PV + storage plant will use retired EV batteries of 75,000 kWh residual capacity (45,000 kWh from LFP batteries and 30,000 KWh from lead-acid batteries), with additional storage capacity of 193,600 kWh from new LIBs [39].

This single example renders the pace of innovation in energy storage (and renewable electricity storage in particular), and reinforces the need to broaden and renew the education of energy managers ​[40], particularly in the field of solar energy [41] whose 2018 photovoltaic output in China grew by 50 per cent in one year only, to the outstanding figure of over 177 TWh ​[42].

Regardless of ongoing reports for which, citing data going back to 2010, lithium-ion batteries would be “currently recycled at a meagre rate of less than 5% in the European Union” ​[43], this study not only refers to actual figures for which, globally, 58% of the world's spent LIBs will be recycled only in 2019 ​[6], but also shows evidence of a global boom of LIB industrial recycling lately extending to numerous countries beyond China.

This is not a research policy study but it cannot be omitted to notice how, reflecting global dominance of China's battery manufacturing and recycling industries, most research articles [21, 22, 24, 25, 34, 35 ] on the recovery of valued metals from spent LIBs were financially supported by China's government through the National Natural Science Foundation of China, and through Provinces interested in preventing pollution *and* in supporting the huge new battery manufacturing and battery EV industries.

There is not shortage of lithium (the mineral raw material) ​[44], but there is shortage of highly pure lithium carbonate and lithium hydroxide (the chemicals) as lately shown, for example, by the scarcity of battery grade lithium lately recorded by the Germany's company willing to start large-scale electric bus manufacturing [27].

In brief, the reuse and the recycling of LIBs is no longer an option but an inevitable need for both battery and battery EV manufacturers.

Helping to further streamline and automate the recycling process, the circular economy companies recycling lithium-ion batteries already work with battery makers to adopt easily dismantled product designs, and will shortly uptake the new green chemistry processes lately developed for the green recovery of all valued battery components.

Energy storage in lithium-ion battery is essential to expand the uptake of clean and renewable electricity for all energy needs including and foremost for powering electric vehicles. Providing an updated global perspective on lithium-ion battery reuse and recycling, this study will be useful to scholars, for example to update content of their teaching, as well as to policy makers devising new policies to promote the energy transition [45].

## Declarations

### Author Contribution statement

Mario Pagliaro, Francesco Meneguzzo: All authors listed have significantly contributed to the development and the writing of this article.

### Funding statement

This research did not receive any specific grant from funding agencies in the public, commercial, or not-for-profit sectors.

### Competing interest statement

The authors declare no conflict of interest.

### Additional information

No additional information is available for this paper.
